# Crèche or Nursery School?

**Published:** 1917-10-06

**Authors:** 


### SOME PROBLEMS OF TO-DAY.

### Creche or Nursery School?

By A SOCIAL WORKER.

OUR most pressing educational and medical problem is now the "toddler." The very invention of this word shows that public attention, shifting as it does from point to point, now focusses its searchlight upon the ex-baby in the working man's home and "discovers" it. Straightway the cry arises that the strength of a chain—to vary the metaphor—is only that of its weakest link. Measures have been taken for the school-child and for the infant. But the ex-baby, a sufferer even in the wealthy nursery when its little nose is suddenly "put out of joint," may be lost or damaged in its passage from mother's breast to infant school. Accordingly it is to be swept into the legislative net, and Mr. Fisher's Bill provides for its training, or rather, to use a word that has grown fashionable, "adumbrates" such provision and leaves the world of practical educationists to think out appropriate schemes before Parliamentary discussion begins. We may accept this as the Minister's intention, for in the address given by him on August 11 at Bedford College to the gathering of practical people forming the "New Ideals" Conference, no detail suggestions were offered. The Conference had to adjourn for want of points to discuss.

### WHO SHALL FETCH THE BABIES?

It was therefore with eager attention that Miss Margaret Macmillan was heard when on a later day she suggested the outlines of a scheme founded on her own experience at Deptford. Sir Robert Morant, from the chair, reminded the audience that differentiation of function is a condition of co-ordinated effort, and a blackboard on the platform was inscribed with that distinction between two different things which it is important that we should recognise. That is, as they exist at present, the day nursery or *crèche* receives children of from three months to five years old, is open from early morning, say 6 A.M., till night, and sometimes for the night also. The nursery school is open for school-hours and dinner-hour for children of two or two and a half to five years. The little ones must therefore be fetched away when school ends, a time impossible for a mother who goes out to work. Miss Macmillan depicted her tinies as coming half asleep, or sometimes quite asleep, from 6 A.M in every kind of weather, bringing a dinner of "pieces" or a green apple covered with treacle. Who should receive such toddlers? was once asked, and the significant reply of an eminent person was "An ayah"! Someone who would cuddle and croon and make an atmosphere of laughter, and rest, and orderly, healthy habits. Teachers, she feels, are not taught to be nurses, nor are nurses generally trained to be teachers. The two really need to be combined, and Miss Macmillan wants to see an army of young women, drawn from every class in the kingdom, trained for this purpose, as her own young helpers are trained, as probationers under efficient heads. Miss Macmillan thinks young children should have young attendants, and views the old nurse with disfavour. Perhaps she would agree that physical age and mental fogeyism are not at all necessarily connected.

### A VALUABLE NEW EXPERT CREATED.

We remember the delicious description in "The Roadmender" of people who were, and others who were not, *old* enough to be real playfellows for the little child. If 1,000,000 children are to be taken in under this scheme, 100,000 such girls would be required, and they would be thoroughly well fed, but not salaried until their third year, and be trained by lectures from experts as well as by apprenticeship to their task. We shall all agree that if anything like this number of young women received two years of practical instruction in mothercraft from the age of sixteen, eighteen, or twenty, as might be decided, a good deal would have been done for our national health. And those who persevered would create a new and very valuable kind of expert.

### OPEN-AIR SHELTERS PREVENT COLDS.

As for the school buildings, they would be open-air shelters as used at Deptford, where the managers find that the children do not suffer from cold, though the elders sometimes do. Such shelters might experimentally —the next ten years must be a time of experiment—be set up in towns on those pieces of waste land which are more numerous there than is generally supposed. The children's health, it is interesting to know, has actually been better in winter than in summer under this *régime*. Attention to physical condition, Miss Macmillan considers, furthers the child's educational progress, and this in turn reacts upon his health. Thus the stuffed nose and stiff upper lip prevent the pronouncing of certain letters. We may ask, in passing, whether this last may not sometimes be due to the use of the "comforter"? This difficulty in speech and reading being overcome by a skilful teacher, the use of explosive Bs and Ps may be expected to improve the circulation in nose and lip, and so tend to diminish catarrh and adenoids. The theory is certainly interesting, and we can hardly doubt that instruction of tinies in singing tends to strengthen throat and lungs. The teacher-nurse should be able to pass on these babies into school at six or seven capable of writing and reading. The order is Miss Macmillan's. Most of us will wonder with her how it is found possible to make any normal child spend eight years or so in learning to read, and all its school life in not learning to spell! Health and vigour must be reduced by such stultifying teaching.

### A CHILD'S SURROUNDINGS HIS SCHOOL.

The nurse-teacher, however, of her scheme must be also the teacher-nurse. She will not allow a child to eat his chalks, or to begin his handwork before finger-nails are clean. Miss Macmillan's belief is that, on the same principle which rolls *crèche*-nurse and school-teacher into one new product, the *crèche* itself would automatically develop the school, as has happened in her own experiment. The psychological moment comes when the infant develops the instinct that seeks to know. He becomes the child, and his surroundings are his school. At Deptford one shelter simply grew into a school. Now this is an important point for those who believe that the Divine system which associates children in families where ages are varied, instead of sending them into the world in litters of the same age, has a great purpose. Children need for balanced growth the influences of older and younger ones, of brothers and sisters. If, therefore, the *crèche* life of the toddler encircles the nursery training of the five-year-old, and both can in some way be connected with the real elementary school, right relations would be established.

(Continued on page 20, col. 1.)

☛ Previous articles appeared March 31, April 7, May 5, 19, and 26, and September 15.

### Some Problems of To-day.

*(Concluded from p. 18.)*

Miss Clara Grant, of the interesting Fern Street School Settlement, suggested during the discussion that in course of time the homes of workers might be built round enclosures in which the buildings to receive the children should stand and be within touch of home and mother.

How to Improve Home and Mother.

This sounds a Utopian development requiring wholly new town-planning. But the principle behind the suggestion is to be welcomed, for to most of us there is something ominous in the idea of a general removal of all toddlers from the homes. This is quite a different matter from the needful provision of safe refuge and proper care and training for children of women obliged to go out to work. But we are constantly told that our great need is to improve the home and the mother. How is this to be done if as soon as her child begins to develop it is removed to the care of others? How, to touch on a smaller practical point, is the ex-baby to be taken to the nursery school? Older brothers and sisters convoy little ones at present, but the toddler must be carried or wheeled. How is the mother to leave an infant twice, or four times, a day in all weathers to do this? And will it be compulsory for one whose home is comfortable? Many other difficulties and drawbacks will occur to any one with any knowledge of children, of education, or of the lives of working people. In the slang phrase of the day, it is "up to us" all to think out our next step in saving the nation's children, and the proposal before us does at least offer broad outlines upon which some workable scheme might be framed.

